# Species Identity and Initial Size Rather Than Neighborhood Interactions Influence Survival in a Response-Surface Examination of Competition

**DOI:** 10.3389/fpls.2020.01212

**Published:** 2020-08-12

**Authors:** Zhiqiang Shen, Yuanzhi Li, Zhiyi Chen, Nianxun Xi, Wenqi Luo, Qing He, Songling Liu, Wei Lin, Xianhui Zhu, Suqin Fang, Youshi Wang, Buhang Li, Chengjin Chu

**Affiliations:** Department of Ecology, State Key Laboratory of Biocontrol, School of Life Sciences, Sun Yat-sen University, Guangzhou, China

**Keywords:** abundance proportion, density, initial seedling size, negative density dependence, response surface design, seedling survival, soil properties, species combination

## Abstract

To measure intraspecific and interspecific interaction coefficients among tree species is the key to explore the underlying mechanisms for species coexistence and biodiversity maintenance in forests. Through the response surface experimental design, we established a long-term field experiment by planting 27,300 seedlings of four tree species (*Erythrophleum fordii*, *Pinus massoniana*, *Castanopsis fissa*, and *Castanopsis carlesii*) in 504 plots in different species combinations (six pairwise combinations of four species), abundance proportions (five abundance proportions of two species, i.e. A: B = 1:0, 3:1, 1:1, 1:3, 0:1), and stand densities (25, 36, 64, and 100 seedlings per plot). In this initial report, we aimed to quantify the relative importance of biotic and abiotic factors on seedling survival at the early stage of growth, which is a critical period for seedling establishment. We found that plot-level seedling survival rate was determined by species combination and their abundance proportion rather than stand density. At the individual level, individual survival probability was mainly explained by species identity, initial seedling size, and soil conditions rather than neighborhood competition. Our study highlights that the seedling intrinsic properties may be the key factors in determining seedling survival rate, while neighborhood effects were not yet prominent at the seedling life stage.

## Introduction

Forests are one of the most important ecosystems in maintaining global biodiversity and consequently determine ecosystem functioning and services (Lang et al., 2012; Wang et al., 2012). The tree seedling stage is the period when survival and growth of individuals are most vulnerable to the change of surrounding biotic and abiotic factors. Exploring seedling survival and regeneration is crucial for understanding species coexistence and community dynamics (Kelly and Bowler, 2002; Lutz and Halpern, 2006), and management of forests (Muller-Landau et al., 2008; Liu S. et al., 2016).

The tree seedling survival is known to be affected by its own intrinsic properties (e.g. its species identity and size, Wang et al., 2012) and its local conditions including biotic (e.g. neighbors, Comita and Hubbell, 2009) and abiotic factors (e.g. soil properties, Bai et al., 2012; Pu et al., 2017). A large body of research has demonstrated that seedling survival in natural communities was significantly influenced by species identity and initial size (Muller-Landau et al., 2008; Wang et al., 2012; Kabrick et al., 2015). For instance, Wang et al. (2012) found that seedling survival increased with its initial size. The larger seedlings can acquire reserves to withstand environmental stress and have an advantage over smaller seedlings in competition for light (Uriarte et al., 2004; Thorpe et al., 2010). Furthermore, different species compositions may result in different results, which indicates that the species identity of both target and neighboring saplings could influence sapling growth and survival (Lang et al., 2012; Yang et al., 2017). Besides the intrinsic properties of tree species, many studies have found that focal seedling survival was also strongly influenced by neighborhood individuals, including the number, diversity, size, and identity of neighbors (Comita and Hubbell, 2009; Chen et al., 2010; Zhu et al., 2015). Specifically, seedling survival was lower surrounded by neighbors of more conspecifics than heterospecifics (He and Duncan, 2000; Comita and Hubbell, 2009; Castagneri et al., 2010; Pu et al., 2017), because of stronger intraspecific competition than interspecific competition for resources (Adler et al., 2018). In addition, tree seedling survival was affected by soil nutrients as well (Chen et al., 2010; Record et al., 2016). For example, seedlings were expected to survive better in soils with higher concentrations of total phosphorus and total nitrogen (Wang et al., 2012). Other soil properties including soil organic carbon and soil moisture also had a significant positive effect on seedling survival (Pu et al., 2017), while topographic variables (e.g. elevation, slope, and aspect) did not (Wang et al., 2012).

To date, numerous tree seedling control experiments have been established worldwide with the original objective to test relationships between biodiversity and ecosystem functioning (Bruelheide et al., 2014; Van de Peer et al., 2016; Verheyen et al., 2016; Grossman et al., 2018). Some studies reported that species richness had no effects on individual seedling survival (Healy et al., 2008; Yang et al., 2013; Yang et al., 2017). These experiments have attempted to explore the survival of seedlings of various tree species and forest types and to test the effects of overall biodiversity on seedling performance. However, they failed to measure the relative importance of species composition, neighbor identity, and stand density on seedling survival due to the methodology limitation. Experimental studies that explicitly examined the relative importance of seedling intrinsic properties, its biotic and abiotic surroundings to individual seedling survival are necessary. Teasing apart the individual effects of those factors would substantially help us identify the key drivers of seedling survival.

The response surface experimental design, where both the density and proportion of the studied species are varying, allows predictions of the long-term ecological outcome of competition (Inouye, 2001; Damgaard, 2008). Therefore, it offers a unique opportunity to compare effects of species intrinsic properties, neighbor identity, individual density, and abiotic factors on focal plants (Inouye, 2001). Most previous studies have used this method to quantify the competitive interactions of plants in grasslands (Weigelt et al., 2007; Damgaard and Kjær, 2009; Damgaard and Fayolle, 2010) and of animals in terrestrial and aquatic ecosystems (e.g., invertebrates, Northfield et al., 2011; fishes, Forrester et al., 2006, and amphibians, Anderson and Whiteman, 2015). To date, few experiments have manipulated tree species, reflecting the logistical difficulties of conducting such complex experiments (Hart et al., 2012).

In this study, we grew four subtropical tree species in different species combinations and tested intra- and interspecific competition by manipulating abundance proportions and stand densities using the response surface experimental design. The overall aim of this experiment is to quantify the niche differences and average fitness differences (two key components of the modern coexistence theory) by measuring intraspecific and interspecific interaction coefficients among tree species, and to further explore the underlying mechanisms for species coexistence and biodiversity-ecosystem functioning relationships in forests (Chesson, 2000; Carroll et al., 2011; Chesson, 2018). In this initial report, we attempted to: (1) at the plot level, test the effects of species combination, abundance proportion, and stand density on the seedling survival rate, and (2) at the individual level, quantify the relative importance of seedling intrinsic properties (species identity and size), biotic (neighbors’ size and density), and abiotic factors (soil properties) on seedling survival probability.

## Materials and Methods

### Study Site

The experiment was established in January 2018 in the Heerkou, Fengkai County, Guangdong Province, China (111°49′E, 23°30′N). This region has a subtropical humid monsoon climate, with the mean annual precipitation of 1744 mm. About 79% of the annual rain falls between April and September, with a pronounced dry season from October to March. The mean maximum temperature is 19.6°C, and monthly average temperature ranges from 10.6°C in January to 28.4°C in July (He et al., 2018; Wang et al., 2019).

### Experimental Design

**Figure 1:**
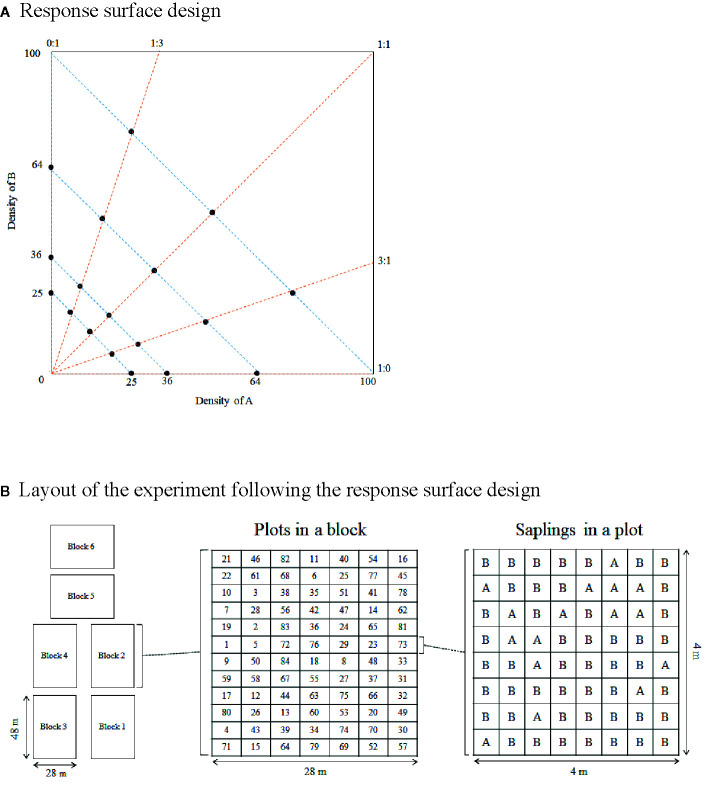
Design of the experiment. **(A)** For each species combination **(A** and **B** for the aim of presentation), 18 treatment combinations of abundance proportion and stand density were considered. **(B)** Each of the six blocks (48 m ×28 m) contains 84 randomly distributed plots (4 m ×4 m). Each plot represents a treatment of species combination, abundance proportion and stand density. The tree seedlings were planted at the equal distance of 0.8, 0.67, 0.5, and 0.4 m, corresponding to the density of 25, 36, 64, and 100 seedlings per plot, respectively.

Our experiment included four tree species: *Erythrophleum fordii* (ERFO), *Pinus massoniana* (PIMA), *Castanopsis fissa* (CAFI), and *Castanopsis carlesii* (CACA), which co-occur naturally in the Dinghushan 20-ha forest dynamics plot, located in a nature reserve near to our experimental site (http://www.efloras.org/flora_page.aspx?flora_id=620; Zhang et al., 2016). Two criteria were set initially in choosing the species: 1) species light strategies, with *P. massoniana* as the light-demanding species, and shade-tolerant species of *E. fordii*, *C. fissa*, and *C. carlesii*; 2) mycorrhizal associations, with arbuscular mycorrhizal species of *E. fordii*, and ectomycorrhizal species of *P. massoniana*, *C. fissa*, and *C. carlesii*. Through these species settings, we plan to quantify the competitive coefficients among species with contrasting light strategies and explore the complementary effects due to mycorrhizal associations on the tree biodiversity-ecosystem functioning relationships. Six pairwise combinations of these four species emerged. Following the response surface experimental design, for each species combination (i.e. A and B), five abundance proportions of two species (A: B = 1:0, 3:1, 1:1, 1:3, 0:1) were set at four different densities (25, 36, 64, and 100 seedlings per plot; **Figure 1A**). This would give us a total number of 120 plots (6 species combinations × 5 abundance proportions × 4 stand densities). However, we did not plant a single species (A: B = 1:0 or 0:1) at the density of 100 seedlings per plot due to the limitation of the site size, which resulted in 108 plots. In addition, 24 more plots with only one species (A: B = 1:0 or 0:1) were redundant across the species combinations involving this species. Ultimately, 84 plots were planted in various combinations of species, abundance proportion and density. We set up six replications (blocks), with each block (48 m × 28 m) consisting of 84 plots with the size of 4 m × 4 m (**Figure 1B**). Overall, we planted 504 (84 × 6) plots with 27,300 seedlings. The tree seedlings were planted at the equal planting distance of 0.8, 0.67, 0.5, and 0.4 m in the densities of 25, 36, 64, and 100 seedlings per plot, respectively. The assignment of plots to treatments was completely randomized, as were the positions of individual tree seedlings within plots.

### Seedlings Planting and Management

The experiment was conducted in an abandoned old field and fenced to exclude large mammalian herbivores in April 2018. Prior to the planting, vegetation in the field was completely removed with a rotary cultivator. Seedlings of the four species were bought from a commercial nursery, which were 1–2 years old ranging from 20 cm to 30 cm height. The seedlings were placed in a nearby temporary shade house to reduce the transpiration. Each seedling was planted in a hole (30 cm × 30 cm × 20 cm), and was watered every day for a week after planting to improve its survival probability. All seedlings were planted according to above planting scheme during April and May in 2018. Drainage channels were set around the blocks to prevent the seedlings from being drowned because of the intensive precipitation in summer. In October 2018, all dead seedlings during the 5 months were replanted. Each seedling was tagged with a unique number specifying its identity. The herbaceous vegetation was cleaned twice a year to reduce their effects on planted seedlings. All the cleanings were manual without using any herbicides.

### Survival Monitoring

In December 2018, 27,029 seedlings were alive during the first survey of survival rate. For each seedling individual, the stem diameter at 5 cm above ground (hereafter ground diameter) were measured as the seedling initial size. Ground diameter was measured with a caliper to the nearest millimeter, and the position of the diameter measurement was permanently marked on the stem with white paint. The survival status of all seedlings was investigated in December 2019, living seedlings were coded as ‘‘1’’ and dead ones as ‘‘0”. In total, 4,130 individual trees were dead during our second survival survey.

### Soil Properties

In December 2018, five soil cores at a depth of 0–10 cm were sampled and mixed for each 4 m × 4 m plot to estimate its average soil conditions. The soil samples were sent to the Institute of Botany of the Chinese Academy of Sciences for chemical analyses, including pH, organic carbon (C), total nitrogen (TN), available nitrogen (AN), total phosphorus (TP), and available phosphorus (AP), which were analyzed according to Lu (1999). We measured the bulk density (BD) and field capacity (FC) of each plot through the ring knife method (Sun Y. et al., 2017). To reduce collinearity of soil factors, principal components analyses (PCA) were conducted on these soil variables. The first two components (PC1 and PC2) accounted for 66.7% of the total variance in soil variables and were used in the subsequent analyses. The first principal component (PC1) was mainly associated with low C, TN, AN, TP, and C/N ratio, and the second principal component (PC2) characterized by low FC, high BD, and pH (**Table S1**).

### Statistical Analyses

We first conducted ANOVAs to explore the effects of species combination, abundance proportion, stand density, and their interactions on survival rates of seedlings at the plot level.

To further explore what factors determined individual survival of these seedlings, we modeled individual survival using a logit link function with a generalized liner mixed model (GLMM) of the general form (a FULL model):

logit (seedling survival) ∼ seedling size + PC1 + PC2 + Scon + Shet + (1|species) + (1|plot)

In this FULL model, seedling size is the initial ground diameter of seedlings. Scon and Shet are the sum of ground diameter of conspecific and heterospecific seedlings within 1 m radius of the focal seedling, respectively. PC1 and PC2 are the first two components of the total variance in soil variables. Species and plot were modeled as random effects. The variables used in the models were summarized in **Table S2**. All continuous explanatory variables were standardized to have zero mean and unit standard deviation prior to the statistical analyses (Bai et al., 2012).

To test the relative importance of seedling initial size, biotic, and abiotic variables, four candidate models were constructed: (1) a NULL model, including seedling ground diameter as only fixed effect; (2) a BIOTIC model, in which the fixed effects of seedling neighbors were added to the NULL model; (3) an ABIOTIC model, in which the fixed effects of soil properties were added to the NULL model; and (4) a FULL model, as described in the above equation, in which all fixed effects of variables were included in the NULL model. Models were compared using the Akaike’s information criterion (AIC) to identify the best fit models, and ones with an AIC value < 2 were considered to be equally valid (Burnham and Anderson, 2002). The variance explained by fixed factors was included in marginal *R* ^2^ (*R* ^2^_mar_) and that by both fixed and random factors was in conditional *R* ^2^ (*R* ^2^_con_) of the models (Nakagawa and Schielzeth, 2013).

To measure the partial effect of each variable on the odds of survival, we calculated odds ratios for each coefficient (the exponential of the estimate of each coefficient). Odds ratio >1 indicates a positive effect on survival, while ratio <1 indicates a negative effect. All data analyses were carried out with the software R version 3.5.3 (http://www.R-project.org).

## Results

### Plot-Level Survival

**Figure 2:**
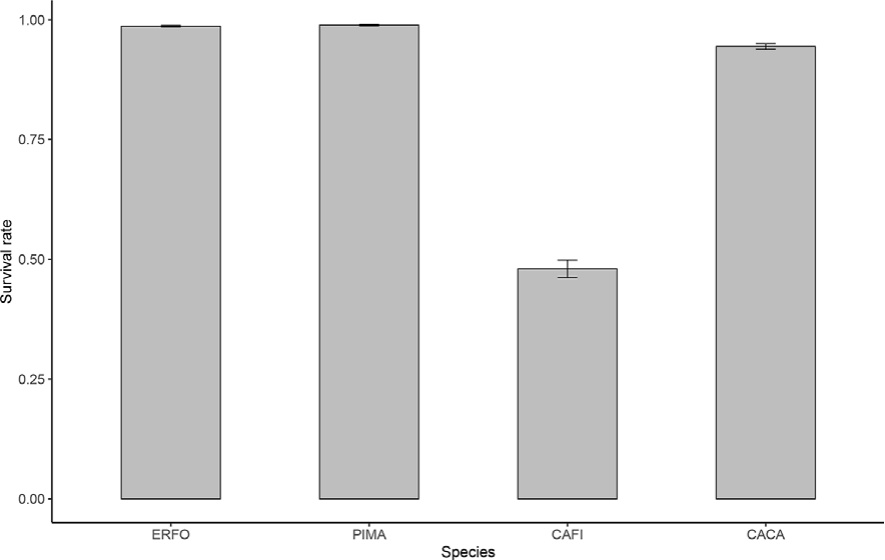
Means and standard errors of seedling survival at the plot level for *E. fordii* (ERFO), *P. massoniana* (PIMA), *C. fissa* (CAFI), and *C. carlesii* (CACA).

**Figure 3:**
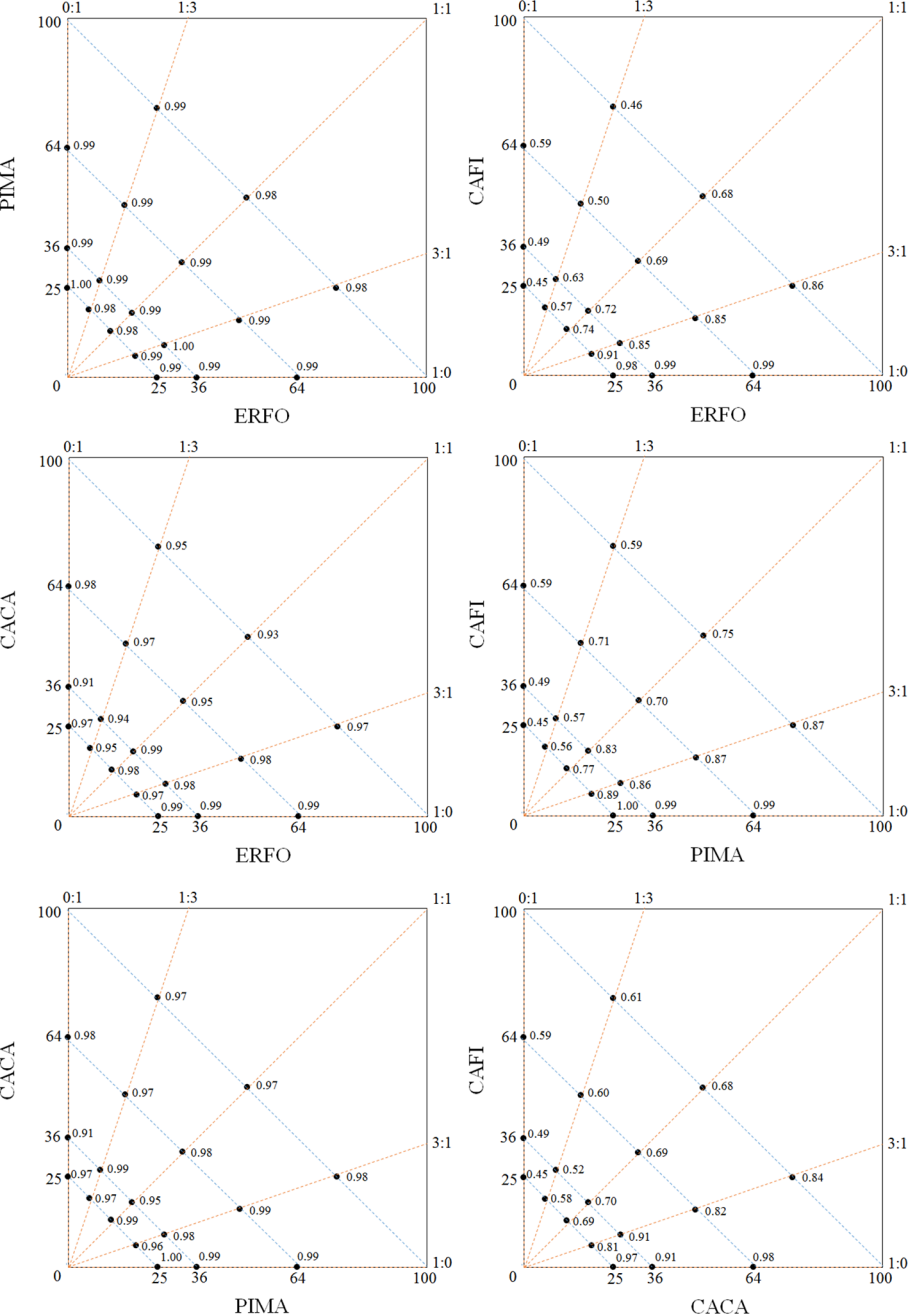
Survival rates of the different treatments. *E. fordii* (ERFO), *P. massoniana* (PIMA), *C. fissa* (CAFI), and *C. carlesii* (CACA).

A total of 27,029 seedlings were alive after the second planting in December 2018 and 22,899 seedlings survived to December 2019. The mean plot survival rate across species was 84.7%, and the mean species survival rates were 98.7%, 98.9%, 48.0%, and 94.4% for *E. fordii*, *P. massoniana*, *C. fissa*, and *C. carlesii*, respectively (**Figure 2**). The plot-level survival rates were significantly affected by species combination and abundance proportion as well as their interactions, but not affected by stand density or its interaction with species combination and abundance proportion (**Table 1**). Specifically, plots of species combination with higher proportion of *C. fissa* had lower survival rates (**Figure 3**).

**Table 1:** ANOVA results for the survival rates at the plot level.

Variable Name	*df*	*F*	*P*
Combination	5	134.068	**<0.001**
Abundance proportion	4	13.424	**<0.001**
Stand density	1	0.020	0.889
Combination × abundance proportion	20	27.108	**<0.001**
Combination × stand density	5	0.549	0.739
Abundance proportion × stand density	4	1.758	0.136
Combination × abundance proportion × stand density	20	0.594	0.918

Significant values are highlighted in bold.

### Individual-Level Survival

**Figure 4:**
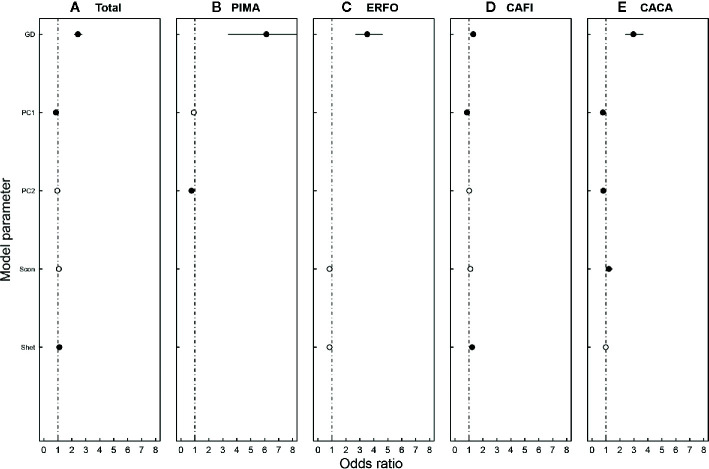
Odds ratios of variables on seedling survival by the best-fit models for **(A)** all species, **(B)** *P. massoniana* (PIMA), **(C)** *E. fordii* (ERFO), **(D)** *C. fissa* (CAFI), and **(E)** *C. carlesii* (CACA). Circles show odds ratios for each parameter, with 95% confidence limits indicated by horizontal lines. Black solid circles indicated significant effects (*P* < 0.05). Variable abbreviations: GD, ground diameter; Scon, sum of ground diameter for conspecific seedlings within 1 m radius; Shet, sum of ground diameter for heterospecific seedlings within 1 m radius.

The individual-level survival including all the four species was best modeled (lowest AIC) by the FULL model considering both biotic and abiotic factors. The fixed factors (seedling ground diameter, biotic neighbors, and soil properties) explained 9.4% of the variance (**Table 2**). Seedling size and heterospecific neighbors had significantly positive effects, while PC1 of soils had negative effects on survival probability of focal seedlings indicated by the odds ratios (**Figure 4A**).

**Table 2:** Effects of biotic and abiotic variables on survival rates of seedlings in December 2019.

Data subsets	Candidate models											
	NULL model			BIOTIC model			ABIOTIC model			FULL model		
	AIC	*R* ^2^_mar_(%)	*R* ^2^_con_(%)	AIC	*R* ^2^_mar_(%)	*R* ^2^_con_(%)	AIC	*R* ^2^_mar_(%)	*R* ^2^_con_(%)	AIC	*R* ^2^_mar_(%)	*R* ^2^_con_(%)
Total	12,022.9	8.6	63.3	12,020.8	8.8	63.6	12,007.4	9.0	62.9	**12,003.1**	9.4	63.3
Species												
*Erythrophleum fordii*	**923.4**	29.0	36.2	**922.4**	30.0	35.5	926.4	29.4	36.0	925.9	30.4	35.3
*Pinus massoniana*	743.5	41.9	56.0	745.6	42.2	57.0	**739.9**	43.8	55.5	742.5	43.9	56.4
*Castanopsis fissa*	7,649.0	1.4	38.2	7638.2	2.3	39.2	7,745.5	2.2	37.9	**7,632.8**	3.2	38.8
*Castanopsis carlesii*	2,500.5	19.4	44.2	2501.0	20.1	44.3	**2,474.3**	23.4	42.4	**2,473.3**	24.2	42.4

The best-fit models are highlighted in bold.

For each individual species, the best-fit model for *E. fordii* was the BIOTIC model, explaining 30.0% of the total variance by fixed factors (seedling ground diameter and biotic neighbors). The best-fit model for *P. massoniana* was the ABIOTIC model explaining 43.8% of the total variance by the fixed factors (soil properties). In contrast, the best-fit models for *C. fissa* and *C. carlesii* were the FULL models, with the fixed factors (seedling ground diameter, biotic neighbors, and soil properties) explaining 3.2% and 24.2% of the total variances (**Table 2**).

The effects of seedling size and abiotic and biotic factors on seedling survival varied among species. Seedling size had the most consistent effects on survival across all species: tree size had the strongest positive effect on survival of seedlings (odds ratio > 1.00, *P* < 0.001; **Figures 4A–E**). There were no significant effects of neighbors on the survival of *E. fordii* seedlings. However, there was a significant positive effect of heterospecific neighboring seedlings on the survival of *C. fissa* (odds ratio = 1.23, *P* < 0.001; **Figure 4D**). Odds ratios for the parameters of the most likely model for seedlings also showed a significantly positive effect of neighboring conspecific seedlings on the survival of *C. carlesii* (odds ratio = 1.21, *P* = 0.033; **Figure 4E**). Soil PC1 showed a marginally negative effect on the survival of *C. fissa* and *C. carlesii* seedlings (odds ratio = 0.86, *P* = 0.002 for *C. fissa*; odds ratio = 0.78, *P* < 0.001 for *C. carlesii*; **Figures 4D, E**). Furthermore, soil PC2 had a significantly negative effect on the seedling survival for *P. massoniana* and *C. carlesii* (odds ratio = 0.76, *P* = 0.011 for *P. massoniana*; odds ratio = 0.81, *P* = 0.004 for *C. carlesii*; **Figures 4B, E**).

## Discussion

### Effects of Species Combination, Abundance Proportion, and Stand Density on Seedlings Survival at the Plot Level

At the plot level, we found that species combination and abundance proportion played an important role in seedling survival in our experiment (**Table 1**). Previous studies showed that species composition affected the growth patterns and crown architecture of tree seedlings (Lang et al., 2012). This indicates that the neighbor tree identity is an important determinant of tree growth (von Oheimb et al., 2011). In our site, when growing together with *E. fordii* and *P. massoniana*, the survival rate of species pair combinations was usually high (**Figure 3**). Conversely, the plots with higher proportion of *C. fissa* generally had a lower survival rate (**Figure 3**). However, the density effects on seedling survival were weak in our study (**Table 1**), in contrast to previous findings (Johnson et al., 2014; Charles et al., 2018). One explanation might be that tree seedlings were too small and distant at the moment to detect significant interactions between them (Plath et al., 2011; von Oheimb et al., 2011). At this stage, these seedlings did not overlap in canopy competing for light and may not have developed extensive root systems competing for soil nutrients (Craine and Dybzinski, 2013; Yang et al., 2017).

### Species Identity and Initial Size Were Drivers of Seedlings Survival at the Individual Level

Consistent with the plot-level survival rate, species identity had a significant impact on individual seedling survival probability (**Figure 4**), which was in line with previous studies in the biodiversity and ecosystem function (BEF) experiments with woody species (Nadrowski et al., 2010; Haase et al., 2015; Peng et al., 2017; Yang et al., 2017). Functional traits play a crucial role in tree seedling survival and growth (Kröber et al., 2015; Li et al., 2017). For instance, plant light-harvesting was significantly affected by branching frequency, leaf distribution, and leaf biomass (Niinemets, 2010; Li et al., 2017). Additionally, energy gain by increased light harvesting may be converted to plant growth and survival, and thus seedling survival and growth were related to species-specific traits (Poorter and Bongers, 2006; Kröber et al., 2015; Li et al., 2017). Wright et al. (2004) reported that the leaf economics spectrum (LES) reflected a mixture of direct and indirect causal relationships between traits. Leaf area plays a central role in leaf trait relationships and can predict tree growth (Osnas et al., 2013). Particularly in young plantations, large-leaved species need more nutrients to quickly increase a stand’s leaf area index, but face greater mortality risk and are more vulnerable. In contrast, species with smaller leaves follow a more invariable investment strategy (Reich, 2014; Kröber et al., 2015). In our site, the survival rates of *E. fordii*, *P. massoniana*, and *C. carlesii* were above 90%, while the survival rate of *C. fissa* was less than 50% (**Figure 2**). This may be due to different growth strategies adopted by species. Specifically, the large leaves of *C. fissa* need more nutrients to achieve higher growth rates, but the root system did not get enough nutrients to support seedling survival. Another explanation for this might be that water would be a limited factor influencing tree survival at the early stage. *C. fissa* has larger leaves, which may lead to a greater transpiration rate. In addition, *C. fissa* is a shade-tolerant species. Previous studies have shown that very shade-tolerant species lack alternative mechanisms to cope with excess light energy and are more likely to incur photoinhibitory damage to their photosynthetic apparatus (Rodríguez-Calcerrada et al., 2006). Therefore, *C. fissa* seedlings had a distinct photoinhibition which reduced carbon assimilation and growth under high radiation in open sites (Yang et al., 2010; Sun Z. et al., 2017).

In agreement with the previous studies (Uriarte et al., 2004; Wang et al., 2012; Pu et al., 2017), we found a significantly positive relationship between initial size and seedling survival (**Figures 4A–E**). The possible reason is that the larger seedlings tend to maintain size advantage over time compared to smaller seedlings (Rose and Scott Ketchum, 2003) partly due to the ability to outgrow competing vegetation (South et al., 2005). In addition, large seedlings are usually in better positions in competition for light (Comita and Hubbell, 2009). However, previous studies also demonstrated that above-ground growth of seedlings did not always indicate the future performance. For instance, the root/shoot ratio has been reported to be directly related to seedling survival in several studies (Lloret et al., 1999; Vaario et al., 2009). The following measurement of root dynamics in our long-term experiment will help us identify the role of below-ground competition on demographic rates of trees.

### Effects of Soil Properties and Neighbors on Seedlings Survival at the Individual Level

In our study, soil nutrients (soil organic carbon, soil total nitrogen, soil total phosphorus, and soil available nitrogen) were all positively associated with individual seedling survival of *C. fissa* and *C. carlesii* (**Figures 4D, E**; Wang et al., 2012; Liu S. et al., 2016; Pu et al., 2017; Yang et al., 2017). The root systems and stems of seedlings could not store more chemical elements at the early stage of the experiment for growth, resulting in more dependence on the available elements in the soil. Therefore, relatively fertile soil would be more beneficial to individual seedling survival of *C. fissa* and *C. carlesii* (Lin et al., 2012; Liu S. et al., 2016). However, the effects of soil nutrients on individual seedling survival rates were not significant for *P. massoniana* and *E. fordii* (**Figures 4B, C**). Previous studies reported that *P. massoniana* was effective at adapting to nutrient-poor soils (Liu J. et al., 2016), which could explain that soil nutrients did not have significant effects on *P. massoniana* seedling survival. Simultaneously, *E. fordii* is a nitrogen fixing tree species. Such characteristics could determine that this tree species has a special ability to improve tree growth and nutrition under nutrient limiting conditions and thus may not depend too much on soil nutrients (Chaer et al., 2011; Mortimer et al., 2013).

Previous studies found that negative density dependence (NDD) plays an important role in driving seedling survival (Comita and Hubbell, 2009; Bai et al., 2012; Pu et al., 2017; Charles et al., 2018). However, we found there was a weak association between conspecific neighbors and focal individual seedlings (**Figures 4A, C, D**). The reason was similar with that of no significant density effects on the plot-level survival rate. Conversely, we found that the focal seedling survival increased as the heterospecific individual seedlings increased (**Figures 4A, D**), indicating facilitative effects of heterospecific neighbors. Our findings are in agreement with previous studies that demonstrated the importance of heterospecific trees for survival of individual seedlings (Peters, 2003; Comita and Hubbell, 2009; Bai et al., 2012; Pu et al., 2017). These are consistent with the species herd protection hypothesis: heterospecific neighbors could depress the encounter probability of focal seedlings and its host-specific enemies and therefore have more benefit to the survival of focal seedlings (Wright, 2002; Peters, 2003; Comita and Hubbell, 2009). In summary, the NDD effect is not very strong at both the plot- and individual-level at the early stage of the experiment.

## Conclusions

Our study presented comprehensive analyses on the relative importance of abiotic and biotic effects on seedling survival, and shed light on the driving factors for species coexistence and community assembly during forest succession. At the plot level, species combination and abundance proportion played an important role in seedling survival rate, while stand density showed little effect on seedling survival at the early stage of seedling planting experiment. At the individual level, species identity, seedling size, and soil properties were more important for seedling survival probability than neighborhood interactions. Although neighborhood interactions had a significant effect on seedling survival (**Figures 4A, D, E**), the effect of NDD was weak. With increasing size of trees, we predict that, as the competition between seedlings strengthens, the effect of the neighbors on the focal tree seedlings would become more pronounced.

## Data Availability Statement

The raw data supporting the conclusions of this article will be made available by the authors, without undue reservation.

## Author Contributions

CC initialized the project. ZS and CC designed the experiment. ZS, ZC, XZ, SL, QH, BL, and WL collected the data. ZS, ZC, and YL analyzed the data. ZS, YL, NX, WQL, SF, YW, and CC interpreted the results and wrote the manuscript. All authors contributed to the article and approved the submitted version.

## Funding

This work was financially supported by the National Key R & D Program of China (2017YFC0506101), and the National Natural Science Foundation of China (31925027, 31622014 and 31570426 to CC, and 31901106 to YL), the China Postdoctoral Science Foundation (2018M643295 to YL), and the Fundamental Research Funds for the Central Universities (20lgpy116).

## Conflict of Interest

The authors declare that the research was conducted in the absence of any commercial or financial relationships that could be construed as a potential conflict of interest.

## Supplementary Material

Table S1 Variable loadings for principal component analyses on soil properties.

Table S2 Parameters used in models of tree survival.
