# Supplementary material for: Species Identity and Initial Size Rather Than Neighborhood Interactions Influence Survival in a Response-Surface Examination of Competition
Source: Front Plant Sci. 2020 Aug 12;11:1212. doi: 10.3389/fpls.2020.01212 (PMC7434863; doi:10.3389/fpls.2020.01212)
Supplement: Table S1 — Variable loadings for principal component analyses on soil properties. [file Table_1.docx]

**Supplementary materials**

**Table S1** Variable loadings for principal component analyses on soil properties.

| Soil variable | PC1 | PC2 |
| --- | --- | --- |
| Field capacity (FC) | -0.274 | -0.543 |
| Bulk density (BD) | 0.199 | 0.598 |
| Organic carbon (C) | -0.469 | 0 |
| Total nitrogen (TN) | -0.448 | 0 |
| available nitrogen (AN) | -0.385 | 0 |
| Total phosphorus (TP) | -0.385 | 0.310 |
| Available phosphorus (AP) | -0.154 | 0.203 |
| pH | -0.124 | 0.434 |
| C/N ratio | -0.359 | 0.124 |
| Variation explained | 45.4% | 21.3% |

**Table S2** Parameters used in models of tree survival

| Parameter | Range | Mean | Median |
| --- | --- | --- | --- |
| Seedling size | | | |
| Seedling ground diameter (GD, cm) | 0.13-2.64 | 0.60 | 0.51 |
| Biotic effect (sum of ground diameter of seedlings within 1 m radius) | | | |
| Conspecific (Scon, cm) | 0-23.51 | 4.16 | 3.46 |
| Heterospecific (Shet, cm) | 0-22.30 | 3.36 | 2.74 |
| Abiotic effect | | | |
| PC1 | -5.48-4.34 | 0.04 | -0.03 |
| PC2 | -4.19-3.97 | -0.01 | 0.11 |
